# Self-delivered arousal-heightening stimuli to improve mobility in persons with parkinsonism: A case series

**DOI:** 10.1177/1877718X251409029

**Published:** 2025-12-22

**Authors:** Gijs Vissers, Anouk Tosserams, Bastiaan R Bloem, Jorik Nonnekes

**Affiliations:** 1 Center of Expertise for Parkinson & Movement Disorders, Department of Rehabilitation, Donders Institute for Brain, Cognition and Behavior, Radboud University Medical Center, Nijmegen, The Netherlands; 2 Center of Expertise for Parkinson & Movement Disorders, Department of Neurology, Donders Institute for Brain, Cognition and Behavior, Radboud University Medical Center, Nijmegen, The Netherlands; 3 Department of Rehabilitation, Sint Maartenskliniek, Ubbergen, The Netherlands

**Keywords:** parkinsonism, arousal, freezing of gait, mobility improvement, kinesia paradoxa

## Abstract

Classic accounts of ‘kinesia paradoxa’ typically involve highly stressful or life-threatening situations, which are difficult to translate to daily life. Here, we describe two patients with parkinsonism who successfully apply self-induced ‘arousal-heightening’ strategies to improve their mobility. The first patient uses an electronically modified fly swatter to self-deliver a mild electrical shock, enabling him to overcome episodes of freezing of gait. The second patient uses his phone's alarm to alert himself, resulting in improved sit-to-stand transfer. These observations suggest that self-induced arousal-heightening strategies represent a hitherto underexplored compensatory strategy that could help to improve mobility in some individuals with parkinsonism.

**Plain language summary title:**

Helping people with Parkinson's disease move more easily using alertness tricks.

## Plain language summary

People with Parkinson's disease sometimes experience sudden freezing, making it hard to move. In life-threatening situations, some people can temporarily overcome this, but these situations are rare and not part of daily life. We describe two people with Parkinson's disease who found their own ways to increase alertness and improve movement. One patient uses a mild electric shock from a modified fly swatter to help him walk when frozen. Another uses his phone alarm to help stand up from a chair more easily. These examples show that self-created ways to increase alertness might be a useful strategy to help people with Parkinson's move more easily.

## Introduction

Gait impairments, and mobility impairments in general, are common and debilitating in PD and atypical parkinsonism.^1^ Patients typically experience a general worsening of motor symptoms, including gait impairment, under circumstances in which they feel stressed or anxious.^2,3^ The detrimental effect of stress and anxiety on the frequency and severity of freezing of gait (FOG) episodes, during which patients feel as if their feet are ‘glued to the floor’,^4^ has been particularly well-established.^5,6^ Consequently, targeted interventions aimed at counteracting stress or anxiety in an effort to enhance walking ability have received increasing attention in recent years.

Paradoxically, a minority of patients actually appear to benefit from stressful or high-arousal experiences.^7^ This is most apparent during ‘kinesia paradoxa’: a sudden, transient ability to carry out a task that a person was previously unable to perform, often in the face of a grave, life-threatening event.^8–10^ A striking example of this took place in the Italian town of L’Aquila, during which patients with PD who had been bedridden for years suddenly managed to escape their nursing home when the town was hit by an earthquake.^9^ Another remarkable example involved a person with advanced PD and severe bradykinesia who was able to quickly get out of bed and run away while experiencing a nightmare.

Although extreme, frightful or life-threatening situations can sometimes transiently improve mobility, deliberately provoking such circumstances is neither practical nor ethical in daily life. As a safer and more feasible alternative, we describe two patients who spontaneously used self-initiated arousal-heightening strategies to enhance their mobility. The first patient uses an electronically modified fly swatter to self-deliver a mild electrical shock, enabling him to overcome episodes of FOG. The second patient uses his phone's alarm to alert himself, resulting in improved sit-to-stand transfer. What is unique in these two cases is their self-discovered use of arousal modulation as a means of inducing kinesia paradoxa. We discuss the possible underlying mechanisms and potential implications for wider dissemination into clinical practice.

## Case 1

**Figure 1.**
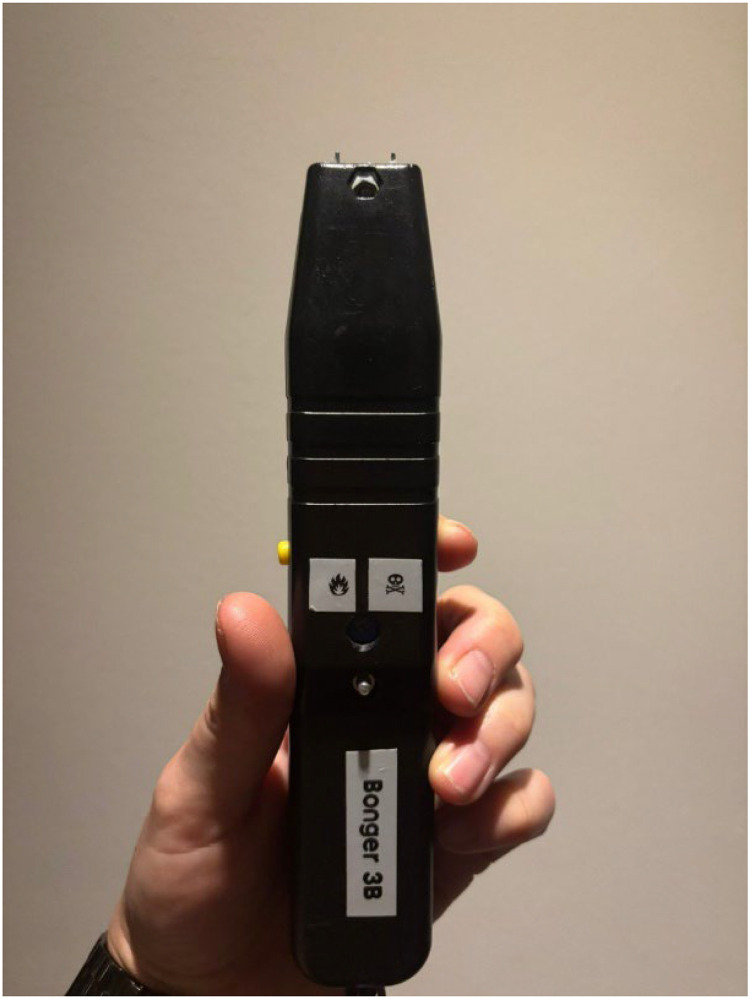
*View of the modified electrical fly swatter (case 1).* At the top, there are two pins through which the patient can self-deliver a mild electrical shock. To activate the shock, the patient presses the button located on the left side of the device. At the front, there is a rotary knob to adjust the intensity of the shock. Turning the knob to the left delivers a milder shock (indicated by the flame symbol), while turning it to the right increases the intensity (indicated by the skull symbol).

This concerns a 67-year-old man initially diagnosed with PD at age 59. Due to a poor response to dopaminergic treatment, a relatively rapid progression of symptoms and marked hypophonia, his diagnosis was changed to MSA-p at age 62. He experienced FOG on a daily basis, with episodes lasting several seconds up to 60 min, and occurring on average 20 times per day, with no observed response to dopaminergic treatment. His FOG episodes were usually triggered by turning and gait initiation. He used cueing and weight-shifting as compensatory strategies, although these had only limited effect. As a result of these FOG episodes, he fell several times per week. Additionally, he experienced frequent daytime sleepiness. He and his family did not report any symptoms that were indicative of a loss of initiative or anxiety. Fortunately, he was mostly able to escape from a freezing episode with the help of his wife, who would usually push him forward to get him going again. However, if he was alone, without his wife around to assist him, he would remain frozen for minutes on end. This eventually led the patient to reach out to a family friend, a retired medical technology developer. He jokingly said to him: ‘If I were to urinate on an electrical wire, that would definitely startle me and get me going. Can you help me with that?’. This inspired his friend to advise the patient's wife to secretly go out and buy an electric fly swatter. When the wife returned home, and found her husband stuck in a FOG episode, she struck him on the leg with the fly swatter - unannounced. This immediately terminated the FOG episode. Following this unexpected but positive first experience, the patient and his friend worked together to optimize the tool for continued daily use. The fly swatter was electronically modified to provide a ‘more humane’ electrical stimulus of which the intensity of the stimulus could be adjusted, without compromising its startling effect. Additionally, it was redesigned to be more manageable to carry around (Figure 1). The patient has been successfully using this self-developed tool on a daily basis for over a year, with a consistent and instant effect, thus far without any indication of habituation (Video 1). With this repurposed electric fly swatter he can now self-deliver this stimulus during a FOG episode by providing the stimulus on his thigh or, for a more intense stimulus, by stroking his index finger. Because of this, he is no longer dependent on the presence of his wife to be relieved of his debilitating episodes. Notably, he reports that even the mere act of carrying the tool around leads to fewer episodes of FOG. Taken together, this has subjectively significantly reduced his daily time spent frozen.

## Case 2

This concerns a 71-year-old man, diagnosed with PD at the age of 56 years. His most prominent symptom was OFF-state FOG. He experienced daily episodes of FOG, particularly when navigating through narrow spaces and during turning. He employed cueing strategies to alleviate symptoms, although their overall impact remained limited. In addition, he often experienced considerable difficulty rising from his chair. His most prominent non-motor symptom concerned a loss of initiative, which manifested as a lack of motivation to engage in household tasks and maintain social contacts with friends and family. Over time, he noticed that he was able to stand up in a more swiftly manner, when he had to rush to get to the front door when the doorbell suddenly rang. He experienced a similar improvement in his ability to rise from his chair when his phone rang and he had to hurry to get to it in time. In his own words, it was the startling effect of the sound, in combination with a sense of urgency brought about by the time-pressure of the situation that propelled him into action. This observation inspired him to experiment with an alarm on his phone. Whenever he struggled to get up from his chair or when a freezing episode occurred, he would set the timer on his phone to a few seconds and turn the volume up. The sound of the alarm would then visibly startle him and facilitate his movements (Video 2). This man used this compensatory trick for several years, without any signs of habituation.

## Discussion

Both patients described here successfully used arousal-heightening strategies to improve mobility. The efficacy of these compensatory strategies can be explained by several underlying mechanisms. We primarily hypothesize that the effectiveness may stem from an increase in arousal, modulated by the noradrenergic system.^11,12^ A recent publication proposed a U-shaped relationship between arousal and gait: increasing arousal improves performance up to an optimal point, beyond which it declines.^7^ Depending on where an individual resides on this curve—what might be considered their ‘baseline position'—an increase in arousal may either improve or worsen their gait. The presented individuals likely had had a suboptimal low state of arousal at baseline: there was excessive daytime sleepiness in the first person, and lack of initiative in the second.

The stimulus likely created an increased and more optimal state of arousal,^13–15^ and this may have impacted gait control in two ways. Gait control partly depends on a locomotor network comprising spinal central pattern generators, mesencephalic and cerebellar locomotor regions in the brainstem, and corticostriatal inputs projecting to the primary motor cortex.^16,17^ Additionally, distributed cortical areas—particularly the frontoparietal and supplementary motor regions—are involved in the continuous adjustment and adaptation of walking.^18^ When walking is performed in an automated manner (i.e., without conscious attention), individuals with Parkinson's disease (PD) often show impaired recruitment of cortical motor areas.^19^ Recent evidence indicates that the noradrenergic ascending arousal system plays a pivotal role in regulating the extent to which these motor circuits receive modulatory input from other cortical regions.^20–22^ Under conditions of suboptimal arousal, integration between distinct and segregated brain networks may be reduced; such integration could potentially be enhanced by strategies that increase arousal.^7^ A second explanation is related to the fact that it is now widely recognized that locomotion is governed not by a single pathway but by multiple, partially parallel locomotor circuits that converge at the midbrain locomotor region—namely, a ventral (emotional) locomotor system and a dorsal (cognitive) locomotor system.^23,24^ Activation of affective circuits may induce a functional shift from the cognitive to the emotional locomotor system.^25^ Importantly, alternative, perhaps even concurrent mechanisms may also be at play: it has been proposed that paradoxical kinesia may be associated with a sudden increase in release of striatal dopamine.^26^

Interestingly, the first patient reported that simply having his electrical fly swatter with him reduced the occurrence of FOG. This is reminiscent of previous accounts of patients who experienced similar improvements from merely having a compensation strategy at their disposal, as this provided them with a backup plan in case of gait difficulties, thereby reducing anxiety.^26^ For example, walking with laser shoes significantly improved one patient's walking ability, even though he did not actively focus on the projected laser beams, simply because he felt more confident.^27^ It is possible that the effect of merely carrying the fly swatter is achieved through the same principle.

Both patients utilized a creative and innovative approach to address their mobility impairments. The question is whether a similar arousal-heightening strategy might also help to enhance mobility in other individuals with PD or atypical parkinsonism. This deserves further study in a broader population. Since we expect that it is the arousal-enhancing effect of the strategies that improved mobility in these patients, we anticipate that any sensory stimulus capable of increasing arousal could potentially serve as an arousal-enhancing strategy. For example, in addition to the tactile and auditory stimuli we presented, also visual stimuli have been reported as triggers of paradoxical kinesia.^10^ Pending the arrival of such additional evidence, we encourage healthcare professionals to consider integrating arousal-heightening into their overall repertoire of compensatory approaches for gait and mobility impairments. Arousal-heightening stimuli are unlikely to be universally effective, but might be helpful for occasional other patients. The two individuals presented here suggest that a arousal-heightening strategy might be specifically worth exploring in patients who report a lack of initiative, and in whom other, more conventional compensation strategies have failed. Future research should also evaluate how these strategies impact on arousal, and explore the neurobiological mechanisms underlying the effect on mobility. Such insights could help to validate the proposed framework. One possible approach is to record physiological markers of arousal (such as heart rate or pupillometry), both with and without the stimulus. Also, subjective reports of arousal during the application of the strategies and during rest were not assessed, which should be considered in future studies. Other work could also focus on developing customer-friendly ways of delivering arousal-heightening stimuli. Interestingly, the effect of the arousal-heightening stimuli did not appear to habituate in both individuals, and this is another element that is worthy of further exploration. Finally, it is worth exploring which specific patient characteristics—like trait anxiety, lack of initiative, or depression—may predict the effectiveness of these strategies, as a basis for personalized treatment.

## Supplemental Material

**Figure:** Video 1.

**Figure:** Video 2.
